# Marine Citizen Science and the Conservation of Mediterranean Corals: The Relevance of Training, Expert Validation, and Robust Sampling Protocols

**DOI:** 10.1007/s00267-023-01913-x

**Published:** 2023-12-16

**Authors:** Laura Figuerola-Ferrando, Cristina Linares, Yanis Zentner, Paula López-Sendino, Joaquim Garrabou

**Affiliations:** 1 Departament de Biologia Evolutiva, Ecologia i Ciències Ambientals, Universitat de Barcelona (UB), Barcelona, Spain; 2 Institut de Recerca de la Biodiversitat (IRBio), Universitat de Barcelona (UB), Barcelona, Spain; 3 Departament de Biologia Marina, Institut de Ciències del Mar (CSIC), Barcelona, Spain

**Keywords:** Marine Citizen Science, Marine conservation, Octocorals, Protocol validation, Volunteer data quality, Mediterranean Sea

## Abstract

Marine Citizen Science (MCS) has emerged as a promising tool to enhance conservation efforts. Although the quality of volunteer data has been questioned, the design of specific protocols, effective training programs, and data validation by experts have enabled us to overcome these quality concerns, thus ensuring data reliability. Here, we validated the effectiveness of volunteer training in assessing the conservation status of Mediterranean coral species. We conducted a comparative analysis of data collected by volunteers with different levels of expertise, demonstrating improvements in data precision and accuracy with only one training session, thereby achieving values equivalent to those obtained by scientists. These outcomes align with the feedback received from volunteers through a qualitative survey. Finally, we analysed the data generated by volunteers and validated by experts using the developed protocol in the *Coral Alert* project from the *Observadores del Mar* MCS initiative. Our findings highlight the importance of proper training, expert validation, robust sampling protocols, and a well-structured platform to ensure the success of long-term MCS projects. Overall, our results stress the key role MCS plays in enhancing the conservation and management strategies designed to mitigate the ongoing environmental crisis.

## Introduction

Citizen science is a growing practice in ecology (Dickinson et al. 2012; Brown and Williams 2019) in which scientists and citizens collaborate to produce new knowledge and achieve learning outcomes that benefit science and society (Kullenberg and Kasperowski 2016; Vohland et al. 2021). Citizen science has been recognized as a new innovative data source that can be included in the Sustainable Development Goals (SDGs) framework (Fritz et al. 2019). This acknowledgement is facilitating the integration and implementation of policy standards and has led to the development of principles in citizen science (Robinson et al. 2019), including specific principles for the marine realm (Garcia-Soto et al. 2017). Marine citizen science (MCS) is a continuously growing field (Thiel et al. 2014; Sandahl and Tøttrup 2020; Garcia-Soto et al. 2021) but remains underrepresented compared with its terrestrial counterparts due to the inherent challenges of sampling in marine environments (e.g. limited access and transportation, diving certification requirement, etc; Roy et al. 2012). The capacity of MCS to span vast temporal and/or spatial scales, reaching areas beyond traditional scientific access, underscores its potential for increasing monitoring and enhancing marine knowledge and conservation efforts (e.g., Brossard, Lewenstein and Bonney 2005; Levrel et al. 2010; Thiel et al. 2014).

While having great potential, citizen science research faces challenges regarding the credibility and quality of volunteer-collected data (Aceves-Bueno et al. 2017), stemming mostly from the potentially higher variability of these data compared to that collected by scientists (Harvey et al., (2002); Moyer-Horner et al. 2012; but see Hoyer et al. 2012; Oldekop et al. 2011). This concern has become a prominent focus in citizen science, leading to recent improvements that include simple and robust sampling protocols, training programs, increased volunteer participation, and larger volunteer groups, among others (e.g., Aceves-Bueno et al. 2017; Lukyanenko et al. 2016). These improvements in data quality strengthen the academic credibility of MCS (Sandahl and Tøttrup 2020) and contribute to the inclusion of citizen science projects in conservation management and policies (Cheung et al. 2022). In addition, the use of volunteer-generated data can transcend beyond presence/absence monitoring (Sandahl and Tøttrup 2020), thus contributing, for example, to the assessment of marine conservation status (Fritz et al. 2019; Kelly et al. 2019, 2020). A representative case of volunteer-collected data in conservation policy is its inclusion into the SDG indicators, which provide a framework for measuring progress towards achieving the SDGs (Fraisl et al. 2020).

To date, MCS studies have mainly focused on documenting species occurrences (Vohland et al. 2021), invasive species (Delaney et al. 2008), marine litter (Locritani et al. 2019), or mass mortality events such as that of the Mediterranean pen shell (*Pinna nobilis*; Cabanellas-Reboredo et al. 2019). The MCS platform *Observadores del Mar* (www.observadoresdelmar.es), launched in 2012, hosts different projects aiming to enhance the conservation of Mediterranean marine ecosystems affected by anthropogenic activities. Among these projects, the *Coral Alert* project studies the distribution and health status of octocoral and hexacoral populations impacted by human disturbances, particularly ocean warming. To assess the conservation status of these populations, the *Coral Alert* project has adopted a cost-effective, straightforward, and robust method called the Mortality Rapid Assessment Method (MRAM; Figuerola-Ferrando et al. 2022), which quantifies the percentage of affected colonies for a specific species, location, depth, and time. Due to its simplicity and easy applicability, this method is suitable for implementation by non-scientific personnel such as managers and volunteers trained through MCS initiatives (see Garrabou et al. 2022a). This information on the population health status complements the valuable knowledge provided by other projects of particular relevance in the Mediterranean Sea, such as the Reef Check Med project, which has collected data on the occurrence, distribution, abundance, and bathymetric range of key marine species along the Mediterranean Sea since 2001 (Turicchia et al. 2021). Considering the concern regarding MCS data quality (e.g., Aceves-Bueno et al. 2017), the aim of this study was to test the reliability of the implementation of the MRAM by recreational divers (i.e., citizen science volunteers), by comparing their data with those obtained by experienced scientists. Specifically, we investigated i) the sampling proficiency of volunteers with different expertise levels compared to that of scientists based on precision and accuracy, ii) the engagement capacity of the *Coral Alert* project and iii) the potential utility of the expert-validated data from the *Coral Alert* project in conservation efforts. Taken together, our results formally validated the MRAM for citizen science, contributing to expanding our knowledge of coral conservation status at large spatiotemporal scales.

## Materials and Methods

### Data Collection

**Fig. 1:**
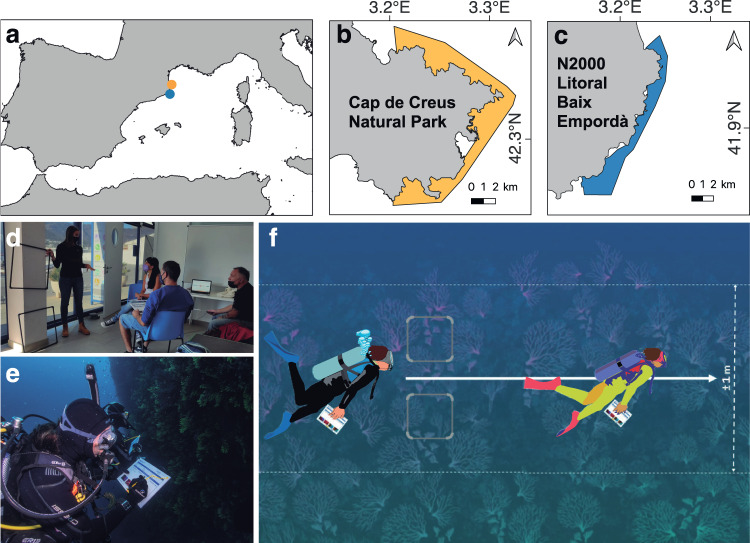
Diagram of citizen science trainings in the northwest Mediterranean (**a**) and specific location of the Cap de Creus Natural Park (**b**) and the N2000 Baix Empordà (**c**). Marine protected areas are shown on the maps in orange (Natural Park, **b**) and blue (Natura 2000, **c**). Images represent a summary of the training, from the theoretical approach (**d**) to protocol application in the field by volunteers (**e**). Diagram of the sampling protocol validation with volunteers, where two marks (50 × 50 cm square) indicate the beginning of the sampling transect (**f**). See Supplementary Fig. S1 for details of the sampling protocol template. Geographic scale and local specific coordinates are indicated for each location (**a**, **b**) in decimal degrees. Symbols are from the integration and application network Library of Symbols, University of Maryland

To validate the MRAM in citizen science, five different trainings were performed along the northwest Mediterranean coast, specifically in Cap de Creus Natural Park and N2000 Baix Empordà (Catalunya, Spain; Fig. 1a–c). In each training, a total of seven to eight volunteers and two to three scientists performed the sampling protocol individually (Fig. 1; see Fig. S1 for details of the sampling protocol template). To assess possible changes in data collection due to the training effort (e.g., improvement in protocol implementation), the same sampling was repeated with the same volunteers a second time in a different training conducted one to three months later. Thus, a volunteer that performed the sampling for the first time was assigned as a “1-day trained volunteer”, while those who repeated the sampling protocol were assigned as a “2-day trained volunteer.” The volunteers’ diving experience was at least 40 logged dives, with a diving certification level ranging from CMAS (*Confédération Mondiale des Activités Subaquatiques*) two stars (46% of the volunteers), to CMAS three stars (33% of the volunteers), to CMAS four stars (professional divers, 21% of the volunteers). Notably, before the campaigns, we ensured that none of the 1-day trained volunteers had previously performed the protocol.

### Volunteer Training and Data Quality Evaluation

The volunteer training to apply the *Coral Alert* protocol consisted of a theoretical session of approximately one to two hours, a practical session of one dive, and a final wrap-up session to discuss the results and address questions regarding the in situ implementation of the protocol. The theoretical session consisted of a presentation given by one of the project’s expert scientist, in which the rationale and the goals of the *Coral Alert* project as well as the MRAM sampling protocol were presented. The MRAM consists of quantifying the percentage of injured coral colonies (or individuals) in approximately 100 colonies, classifying them as non-affected colonies, colonies affected by recent mortality (denuded tissue or necrosis), colonies affected by old mortality (epibiosis), or both (Fig. S1; see Figuerola-Ferrando et al. 2022). For the practical sessions, the red gorgonian (*Paramuricea clavata*) was selected because it is a dominant octocoral and also one of the most affected by climate-driven disturbances in the sampling area. To ensure that all observers (volunteers and scientists) sampled the same population of *P. clavata* at the same location and depth, a reference was used to mark the beginning of the sampling transect. This reference consisted of two squares of 50 × 50 cm situated on the vertical wall (Fig. 1f). Each observer started the sampling protocol from the reference and moved to the same direction and depth (without exceeding a depth of ±1 m) until 100 colonies were reached (Fig. 1f).

The percentage of affected colonies and the total number of colonies sampled by the observer were used as the two variables to validate the sampling proficiency of the citizen science volunteers according to the accuracy and precision of the data. These variables were analysed by comparing the volunteer-collected data with the scientist-collected data, as well as by comparing the volunteer-collected data among themselves (i.e., between 1-day trained volunteers, 2-day trained volunteers, and scientists). To analyse data from different locations, degrees of impact, and sampling days, we first established a reference value for each training. For the percentage of affected colonies, the reference value corresponded to the mean percentage of affected colonies assessed by the scientists in the same training. We then calculated the differences between the values collected by each observer (volunteers and scientists) and their respective reference values. Regarding the total number of sampled colonies, a minimum reference value of 100 colonies was used to validate the ability of the volunteers to apply the sampling protocol. Thus, in this case, we evaluated the ability of citizen science volunteers to sample 100 or more colonies of the same species, location, and depth. The non-parametric Kruskal‒Wallis test (Kruskal and Wallis 1952) was used to test the effect of the three expertise levels on the data collected by the observers (volunteers and scientists) related to the percentage of affected colonies and the total number of sampled colonies.

After assessing the percentage of affected colonies, the MRAM classifies the information into four impact categories, facilitating a rapid evaluation of the conservation status of the monitored populations. These four impact categories based on the values of the mean percentage of affected colonies were: non-impacted population (<10% of affected colonies), low-impacted population (≥10%, <30% of affected colonies), moderately impacted population ( ≥ 30%, <60% of affected colonies), and severely impacted population (≥60% of affected colonies; Figuerola-Ferrando et al. 2022). Thus, as the final step to validate the protocol, the conservation status (i.e., the impact category) determined by volunteers and scientists was compared.

To address the potential risk of bias among volunteers, apart from ensuring their diving buoyancy (i.e., minimum of 40 logged dives), we also confirmed that their diving certification level did not affect the results (see Fig. S2).

### Engagement Capacity Assessment

After sampling, volunteers uploaded their results to the *Observadores del Mar* platform and completed a qualitative survey to assess their satisfaction with the overall process. Volunteers trained for 2 days were asked about their observations between the first and second sampling (yes/no questions). All volunteers were asked whether they observed more gorgonian mortality on their recreational dives after the training (yes/no question). Additionally, we asked all volunteers about the theoretical and practical part of the training, the implementation of the sampling protocol, and the *Observadores del Mar* platform (questions rated from 0 to 10; see Table S2 for details). Note that volunteers had to register beforehand to the platform if they were not already registered in other projects.

### Potential Application for Conservation

To assess the applicability for conservation purposes of the data collected by the volunteers through the MRAM, we conducted a comprehensive review of the data collected in the framework of the *Coral Alert* project. This was possible due to the validation of the use of the MRAM by volunteers in this study. We analysed the geographic and temporal coverage, including both the incidental occurrence observations (i.e., pictures and geographical position of octocorals and hexacorals taken by recreational divers during their dives), and the observations where volunteers had conducted the MRAM. The analysis regarding the conservation status based on the MRAM protocol data was assessed by location and 5-year time period. Locations included different monitoring areas (e.g., the Balearic Islands, the Gulf of Lion, or the Ligurian Sea). The conservation status was represented as the percentage of the samplings that reported each impact category in a specific location across the entire year period.

## Results

### Comparing Data Obtained from Volunteers and Scientists

**Fig. 2:**
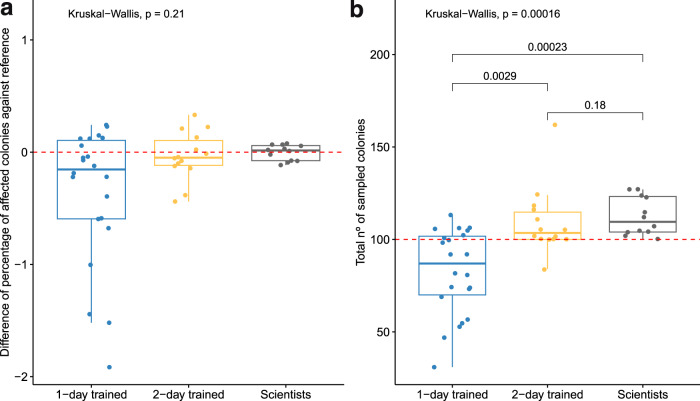
Volunteer sampling proficiency according to precision and accuracy. The percentage of affected colonies in the three expertise levels (1-day trained volunteers, 2-day trained volunteers, and scientists) is expressed as the percentage of difference in affected colonies assessed by observers from the reference value (**a**), which is the mean percentage of affected colonies assessed by scientists in each training. The total number of sampled colonies in the three expertise levels (**b**) is expressed as the total number of sampled colonies by each observer. The horizontal dashed red line represents the reference value in **a** and the minimum number of sampled colonies required (100) to accurately perform the protocol in **b**. *P* values are from a Kruskal‒Wallis test comparing the effect of expertise on the percentage of colonies assessed in a and the total number of sampled colonies in **b**

The application of the MRAM revealed contrasting results between 1-day and 2-day trained volunteers (Fig. 2). Volunteers showed more variability than scientists when quantifying the percentage of affected colonies, as the results given by volunteers showed more differences from each other, especially among the 1-day trained volunteers (Fig. 2a). This result indicated a lower precision (i.e., repeatability of the measurements) among the 1-day trained volunteers but an enhanced precision among the 2-day trained volunteers, approaching levels close to those achieved by scientists. Notably, most of the 1-day trained volunteers assessed a smaller percentage of affected colonies than expert scientists, even reporting values as low as half of the scientists’ values (i.e., values below “−1” in the difference in percentage against the reference; Fig. 2a). In contrast, the percentage of affected colonies was statistically consistent and close to the reference value between each level of expertise (i.e., 1-day trained volunteers, 2-day trained volunteers, and scientists; Kruskal‒Wallis, *X*^*2*^ = 3.14, *p* = 0.21; Fig. 2a). This result indicated high accuracy (i.e., closeness to the real value) at all expertise levels. However, the 1-day trained volunteers showed mean percentage values of affected colonies slightly more distant from the reference value compared to those from 2-day trained volunteers and scientists. Scientists showed better adjustments around the reference, indicating a very low variability in the assessment of the percentage of affected colonies (high precision; Fig. 2a).

The volunteers who performed the sampling protocol twice significantly improved their sampling proficiency in terms of the total number of sampled colonies. The vast majority of them (93%) exceeded the minimum reference value of 100 sampled colonies recommended by the protocol and showed no significant differences from scientists (Kruskal‒Wallis, *p* = 0.18; Fig. 2b). In contrast, the 1-day trained volunteers generally sampled fewer than 100 colonies (only 36% of them exceeded the reference value), leading to significant differences with respect to the 2-day trained volunteers and the scientists (Kruskal‒Wallis, *p* = 0.0029; *p* < 0.001, respectively; Fig. 2b). Notably, some of the 1-day trained volunteers sampled less than half of the recommended value of 100 colonies (i.e., values below “50” in the total number of sampled colonies; Fig. 2b). The potential outlier from a 2-day trained volunteer (with a total of 162 sampled colonies; Fig. 2b) is not an issue, as the protocol does not limit the maximum number of sampled colonies. Indeed, all scientists exceeded the minimum number of sampled colonies, showing a slightly larger dispersion above this minimum threshold (Fig. 2b).

Finally, the impact category assessed by volunteers was the same as the assessed by scientists regardless of the number of trainings attended when using the mean percentage of affected colonies (Table S1).

### Engagement Capacity

**Fig. 3:**
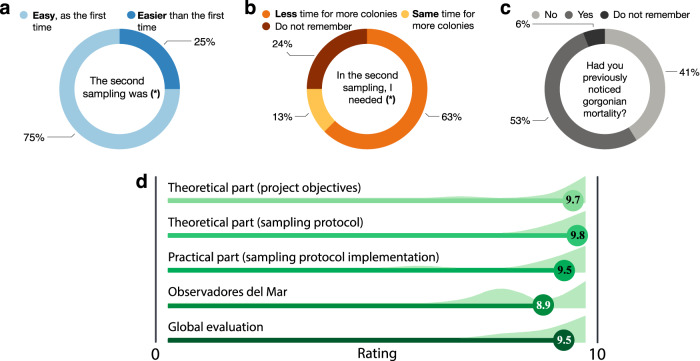
Key findings of the volunteer survey. The response in the first panels (**a**–**c**) is given by a percentage. Panel **d** shows the average value of the response among volunteers, from 0 (strongly disagree) to 10 (strongly agree), where the density of responses is shown above each line. Panel **d** shows a comprehensive summary of the main points of the survey regarding the theoretical session (clarity and level of the contents of the *Coral Alert* project and the information given for the application of the sampling protocol), the practical session, and the valuation of the *Observadores del Mar* marine citizen science initiative (data uploading process and general assessment). Note that questions marked with (*) were only asked to 2-day trained volunteers. To perform Panel **d**, we used an adaptation of mentimeter.com (Iona 2018)

Our quantitative results are in accordance with the volunteers’ impressions measured by a qualitative survey applied after the trainings (Fig. 3; Table S2 for details). All volunteers agreed that applying the sampling protocol was easy, and they felt confident in applying it after 1 or 2 days of training without further expert supervision (Table S2). Among the 2-day trained volunteers, 25% reported that the second sampling was even easier due to the expertise acquired during the trainings (Fig. 3a, b). Moreover, 63% of these volunteers reported completing the sampling faster on their second attempt (Fig. 3b). Regarding the direct impacts on the knowledge of the volunteers, 41% of the 1-day and 2-day trained volunteers acknowledged not having noticed coral mortality before the trainings, despite having dived frequently in coral-dominated assemblages (Fig. 3c). The general satisfaction level with the trainings was high (mean rating 9.5/10; Fig. 3d), including with the theoretical and practical approach, the information given for the application of the sampling protocol, and the *Observadores del Mar* MCS platform (Fig. 3d).

### Potential Application of the *Coral Alert* Project

**Fig. 4:**
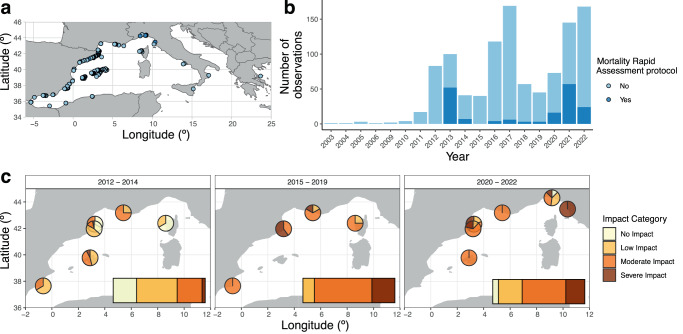
Summary of the data uploaded and validated in the *Coral Alert* project (marine citizen science platform *Observadores del Mar*; www.observadoresdelmar.es) from 2003 to 2022. Geographical (**a**) and temporal (**b**) coverage of incidental observations in light blue and observations including the sampling protocol in dark blue (Mortality Rapid Assessment protocol No/Yes, respectively). The conservation status of gorgonian populations in each year period (**c**) is expressed by a colour scale according to the impact category (non-impacted populations (<10% of affected colonies), low-impacted populations (≥10%, <30% of affected colonies), moderately impacted populations (≥30%, <60% of affected colonies), and severely impacted population (≥60% of affected colonies) and is represented as the percentage of sampling protocol observations reporting each impact category across the entire year period for each location (pie charts) and all locations together (bar plots). The conservation status assessment in **c** includes different species (*Corallium rubrum*, *Eunicella cavolini*, *Eunicella singularis*, and *Paramuricea clavata*)

The geographical extent of the *Coral Alert* project observations is mostly focused on the northwest Mediterranean Sea and includes the countries of Spain, France, and Italy, as well as Algeria and Greece (Fig. 4a). The region with the most observations is the north coast of Catalonia and the Balearic Islands (Fig. 4a), while the most observed species are octocorals, specifically *Paramuricea clavata* and *Eunicella singularis*, followed by the hexacoral *Cladocora caespitosa* (297, 241, and 185 observations, respectively; Table S3). The number of observations included within the *Coral Alert* project has increased during the past almost two decades. The total count stands at 1,068 validated observations, including both incidental occurrence data (84%) and observations utilizing the MRAM protocol (16%). Notably, within the MRAM protocol observations, the sampling effort reached 16,489 colonies (Fig. 4b; Table S3). The years with the most uploaded and validated observations are 2017, 2021, and 2022 (169, 145, and 168 observations, respectively; Fig. 4b).

The observations acquired through the implementation of the MRAM protocol by trained volunteers revealed a worsening of the conservation status of the sampled gorgonian and coral populations over the 2012–2022 period (Fig. 4c). Overall, during the 2012–2014 period (59 observations), volunteers assessed more locations with a higher proportion of non-impacted or low-impacted populations. In contrast, during the 2015–2019 period (16 observations), moderate and severe impacts increased, while the proportion of low-impacted populations decreased. Finally, in the 2020–2022 period (97 observations), sampled populations showed a moderate impact category, with a remarkable increase in severely impacted populations (Fig. 4c). Notably, the latter period had the highest number of observations, including those acquired with the MRAM protocol (Fig. 4b).

## Discussion

Our study successfully demonstrates the reliability of data collected by citizen science volunteers applying the MRAM based on the quantification of the percentage of affected coral colonies or individuals, reinforcing the effectiveness, robustness, and potential application of MCS in marine conservation. Our findings highlight the importance of training, expert validation, and the support of a well-structured platform to ensure the success of long-term citizen science projects, especially those that include underwater sampling protocols.

### Volunteer Improvement and Recommendations

The percentage of affected colonies and the resulting conservation status assessed by the 2-day trained volunteers and scientists were comparable, while the 1-day trained volunteers obtained quite notable results in the first sampling. Data accuracy and precision testing is quite common in MCS studies, which generally employ comparisons of volunteer-generated data with a reference value generated by experts (e.g. Mumby et al. 1995; Evans, Birchenough and Fletcher 2000; Goffredo et al. 2010) or subsequent expert review of samples (e.g., Hidalgo-Ruz and Thiel 2013). Our work expands these outputs to Mediterranean coral species, supporting the strategies and recommendations employed to improve the quality of citizen science data (Wiggins et al. 2011; Freitag, Meyer and Whiteman 2016; Kosmala et al. 2016; Aceves-Bueno et al. 2017). The 1-day trained volunteers did not differ from the 2-day trained volunteers and scientists in terms of the mean percentage of affected colonies assessed. However, the variability among 1-day trained volunteer observers was higher, but some motivated volunteer scuba divers were able to obtain valuable results on the first day of training (e.g., this study; Edgar and Stuart-Smith 2009). In contrast, the precision of the total sampled colonies was significantly lower among the 1-day trained volunteers but was clearly increased after only one sampling experience. Notably, volunteers not only improved their precision when training but also their accuracy by reducing the variability among observers while approaching reference values for both variables. The improvement in data quality over time in citizen science studies is notable. This study, combined with other practical validations (e.g., Pescott et al. 2015; Falk et al. 2019), reinforces the relevance of training, expert validation, and volunteer self-efficacy above other factors such as diving certification level, number of volunteers, dive time, age, or education (Crall et al. 2011, Goffredo et al. 2010; but see Engel and Voshell 2002; Galloway, Tudor and Haegen 2006, Hermoso et al. 2019). Moreover, our study also integrates other essential dimensions in biology-themed citizen science data quality (Lewandowski and Specht 2015), such as the specification of sample size (i.e., minimum number of 100 sampled colonies/individuals), the spatiotemporal representation (i.e., Mediterranean scale; and applied since 2012), and a standardized sampling protocol (MRAM).

The final aim of the MRAM is to easily determine the conservation status of key marine habitat-forming species (e.g., cnidarians, sponges, bryozoans, and calcareous algae) in the Mediterranean Sea in the long term over large spatial scales. It has been successfully applied by scientists to other species of octocorals (*Eunicella cavolini*, *E. singularis*, *Leptogorgia sarmentosa*, and *Corallium rubrum*), hexacorals (*Balanophyllia europaea*, *Cladocora caespitosa*, *Leptopsammia pruvoti*, *Madracis pharensis*, and *Oculina patagonica*), and sponges (*Sarcotragus fasciculatus*; Garrabou et al. 2009, 2022a, b; Crisci et al. 2011; Sini et al. 2015; Kružić et al. 2016; Rubio-Portillo et al. 2016; Betti et al. 2020; Zentner et al. 2023). Thus, these species could be included as targets in citizen science projects. However, the implementation of the MRAM by volunteers should be validated, especially for hexacorals and sponges, for which some specifications can be provided (e.g., reducing the assessment to only partial and total mortality without distinguishing between recent and old injuries; Figuerola-Ferrando et al. 2022). Our results demonstrate that citizen science volunteers are effective at categorizing the same impact category as scientists, albeit with higher variability, especially volunteers trained for only one day. For this reason, we recommend that when the first-time volunteers perform the sampling protocol, they report the average number of affected colonies/individuals of all observers to avoid errors arising from precision and accuracy, whereas after the second day of training, volunteers are sufficiently qualified and skilled to report the results individually or jointly with the diving colleague. This information should be included in the profiles of the registered volunteers to facilitate data processing (it should be noted that this will be one of the next functionalities to be included in the *Observadores del Mar* platform). In addition to the importance of training, it is worth emphasizing the expert validation of the data prior to their transfer and analysis. The validation process is crucial to ensure that the conservation status assessments are consistent and reliable. Moreover, it is proposed that observations including the MRAM protocol be conducted in late summer and/or early fall (September to November) to integrate all the potential recent effects caused by thermal conditions experienced during the summer. Finally, given the vulnerability of the studied habitats and their specific characteristics (i.e., typically occurring on vertical walls at depths greater than 15 m), it is recommended that the minimum certification level to perform this sampling protocol should be at least CMAS two star (or equivalent) with a diving experience of at least 40 logged dives. These recommendations are the result not only of this work but also of the application of the protocol in the *Coral Alert* project.

### Engagement Capacity of the *Coral Alert* Project and Conservation Implications

The survey showed that citizen science volunteers increased their knowledge regarding the effects of global warming on Mediterranean corals. The theoretical part of the training (including the project goals and the information given for the implementation of the sampling protocol) obtained the highest score (9.7 and 9.8 out of 10, respectively; Fig. 3d), while all volunteers were confident and motivated to implement the protocol in the future without supervision (Table S2). Knowledge, together with recognition, are the most rewarding motivational incentives in MCS projects, as they increase the social and conservation awareness of volunteers and are some the most important factors driving the growth of citizen science (Thiel et al. 2014; Campbell and Smith 2006).

Obtaining observations through MCS projects can be challenging, especially when it involves underwater sampling (Goffredo et al. 2010). Our project not only relies on a robust method but also benefits from a well-structured platform, as effective MCS projects require more than just a well-designed protocol (McKinley et al. 2017). Specifically, *Observadores del Mar* encompasses the main pillars of a successful MCS initiative (European Marine Board; Garcia-Soto et al. 2017), emphasizing its support for the generation of scientific knowledge (e.g., Azzurro et al. 2016, 2020, 2022; Cabanellas-Reboredo et al. 2019) and data transfer to international databases (e.g., Chic and Garrabou 2020 in the Global Biodiversity Information Facility, GBIF). In fact, this platform is an open and accessible database (data can be consulted on www.observadoresdelmar.es and downloaded from GBIF), with the capacity to be integrated with other sources and be reusable, in clear alignment with the FAIR (Findable, Accessible, Interoperable, and Reusable) data principles (Wilkinson et al. 2016). *Observadores del Mar* also adheres to the ten principles for citizen science outlined by the European Citizen Science Association (Robinson et al. 2019) and complies with the five dimensions required to integrate an indicator into the SDGs framework (Fritz et al. 2019). Specifically, the *Coral Alert* project started in 2012 and has accumulated over 1,000 validated observations from more than 200 volunteers, spreading its geographical extent from the northwest Mediterranean to southern and eastern areas, involving more countries, observers, and scientists. In recent years, trainings, volunteers, and observations including the MRAM protocol have grown successively along with the number of sentinel observatories—which are organizations such as diving clubs, diving centres, and environmental associations that agree to monitor specific coastal areas ˗ and international projects involving the implementation of this protocol (e.g., MPA-Engage; see Garrabou et al. 2022a). Likewise, in collaboration with recreational diving organizations through the development of marine citizen science programs such the Basic Research Operator by DAN and PADI (https://danrni.eu/progetti/basic-research-operator/), the MRAM protocol is a promising line of action to be explored to expand the number of observations in a consistent way. These synergies reinforce the data quality of the observations, with increasingly trained volunteers in different regions of the Mediterranean, which hints at the great future potential of the project contributing to conservation.

MCS projects hold significant promise for enhancing marine conservation efforts (Earp and Liconti 2020). Here, the overview of the impact category assessed by volunteers in the northwest Mediterranean is in line with peer-reviewed studies (e.g., Garrabou et al. 2022b). Indeed, the 2015–2019 period was considered the warmest on record in the whole Mediterranean Sea, affecting a wide range of different taxa down to 45 m depth (Garrabou et al. 2022b). Unfortunately, these exceptional thermal conditions have become commonplace in recent years (see www.t-mednet.org), and the validation of the MRAM protocol in citizen science, together with others (e.g., Reef Check Med; Cerrano, Milanese and Ponti 2017; Ponti et al. 2020; Turicchia et al. 2021), is increasing our ability to assess their effects. Applying this method through citizen science is not only beneficial to bolster scientific knowledge but can also be used to guide management efforts. Notably, MSC is already being employed to improve marine conservation and legislation (Crabbe 2012; Hyder et al. 2015). Some projects have already contributed to the designation and/or monitoring of marine protected areas (e.g., Earp and Liconti 2020), and others have already been included in different SDG indicators (Fraisl et al. 2020). In this context, the *Coral Alert* project, which directly assesses the conservation status of Mediterranean coral species, represents a strong candidate for consideration.

## Supplementary Information

Supplementary Information

## Data Availability

All data generated or analysed during this study are included in this published article [and its supplementary information files] or available from the Marine Citizen Science initiative *Observadores del Mar* (www.observadoresdelmar.es) upon request.
