# Supplementary material for: Marine Citizen Science and the Conservation of Mediterranean Corals: The Relevance of Training, Expert Validation, and Robust Sampling Protocols
Source: Environ Manage. 2023 Dec 16;73(3):646–56. doi: 10.1007/s00267-023-01913-x (PMC10884135; doi:10.1007/s00267-023-01913-x)
Supplement: Supplementary file 1 — Supplementary Information [file 267_2023_1913_MOESM1_ESM.docx]

**SUPPLEMENTARY INFORMATION**

The supplementary information of the manuscript “**Marine Citizen Science and the conservation of Mediterranean corals: the relevance of training, expert validation and robust sampling protocols**” by Laura Figuerola-Ferrando, Cristina Linares, Yanis Zentner, Paula López-Sendino and Joaquim Garrabou details the specific information of the citizen science trainings and the summary of the data updated in the Coral Alert project (Observadores del Mar; [www.observadoresdelmar.es](http://www.observadoresdelmar.es)).

The specific details of the Mortality Rapid Assessment protocol template used in the citizen science trainings is given in Fig. S1. This protocol template is also being used in the Coral Alert project to assess the conservation status of different marine habitat-forming species.

Table S1 details the impact category obtained by citizen science volunteers and scientists in each training. Note that the expertise of volunteers from each training (1-day trained volunteers or 2-day trained volunteers) is specified.

To ensure that the results were not biased by volunteers diving certificate, we performed a pre-analysis given in Fig. S2.

Table S2 reports the full responses of the survey answered by the citizen science volunteers after trainings. Questions only asked to the 2-day trained volunteers are indicated.

Finally, Table S3 summarizes the data updated by volunteers and validated by experts in the Coral Alert project, which is separated from each species. The total number of validated observations, the total number of sampling protocol validated observations, and the total number of sampled colonies in the sampling protocol observations are reported.

**
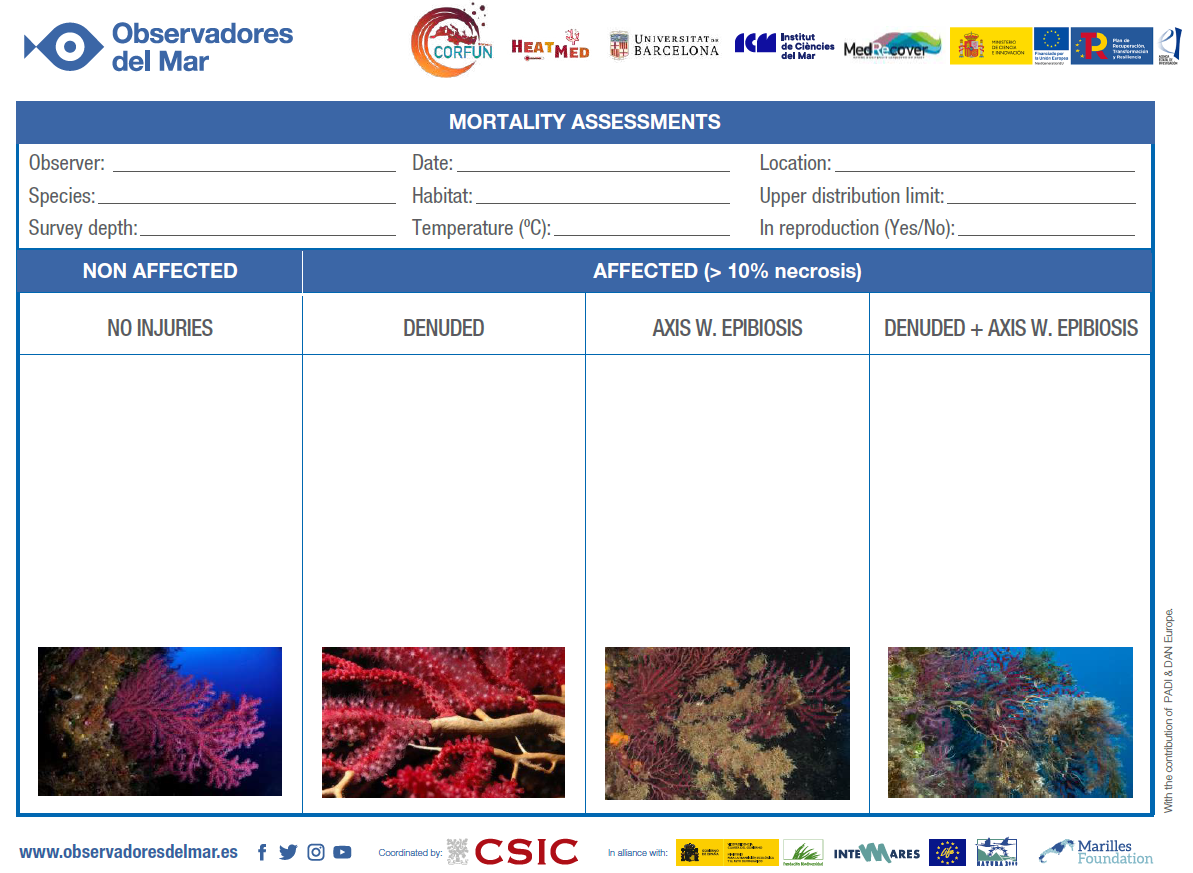
**

**Fig. S1.** Sampling template of the Mortality Rapid Assessment protocol - percentage of affected colonies - from the *Coral Alert* project (*Observadores del Mar* citizen science platform: [www.observadoresdelmar.es](http://www.observadoresdelmar.es)). In the present study, the template was used for citizen science volunteers and scientists to assess the conservation status of gorgonian populations in the sampling trainings and then to validate the results between them.

**Table S1.** Impact category given by scientists and volunteers in each training. The percentage of affected colonies is expressed as the mean ± standard deviation (SD) for each expertise group (1-day trained volunteers, 2-day trained volunteers, or scientists) and training. Impact category is classified into four categories: non-impacted populations (< 10% of affected colonies), low-impacted populations (≥ 10%, < 30% of affected colonies), moderately impacted populations (≥ 30%, < 60% of affected colonies), and severely impacted population (≥ 60% of affected colonies).

| **Training** | **Expertise** | **Percentage of affected colonies (mean ± SD)** | **Impact category** |
| --- | --- | --- | --- |
| Cap de Creus | Scientists | 43.32 ± 4.43 | Moderate |
|  | 1-day trained | 37.00 ± 14.87 | Moderate |
| N2000 Baix Empordà | Scientists | 43.66 ± 5.18 | Moderate |
|  | 1-day trained | 41.40 ± 10.21 | Moderate |
| N2000 Baix Empordà | Scientists | 45.16 ± 1.31 | Moderate |
|  | 1-day trained | 33.80 ± 13.34 | Moderate |
| Cap de Creus | Scientists | 79.03 ± 7.26 | Severe |
|  | 2-day trained | 68.31 ± 8.61 | Severe |
| N2000 Baix Empordà | Scientists | 43.43 ± 2.84 | Moderate |
|  | 2-day trained | 50.19 ± 9.23 | Moderate |

**
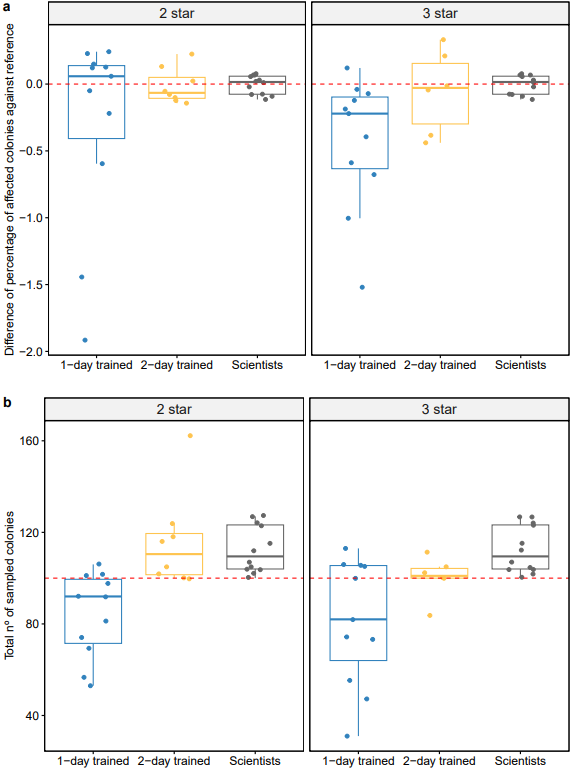
** **Fig. S2.** Certification level (CMAS two star, “2 star”; and CMAS three and four star, “3 star”) comparation. The percentage of affected colonies in the three expertise levels (1-day trained volunteers, 2-day trained volunteers, and scientists) is expressed as the percentage of difference of affected colonies assessed by observers from the reference value (a), which is the mean percentage of affected colonies assessed by scientists in each training. The total number of sampled colonies in the three expertise levels (b) is expressed as the total number of sampled colonies by each observer. The horizontal dashed red line represents the reference value in A, and the minimum number of sampled colonies required (100) to accurately perform the protocol in B. Note than scientists diving certification does not been considered.

**Table S2. Completed results of the survey answered by the volunteers.** All responses are shown as percentages. Note that questions marked with (*) were only asked to 2-day trained volunteers.

| **Number of the question** | **Question and possible responses. (Note than when response is a rating above 0 to 10, only answered responses are included).** | **Percentage of response (%)** |
| --- | --- | --- |
| **1** | **Did you know about the *Observadores del Mar* before the training?** | |
|  | Yes | 82.6 |
|  | No | 17.6 |
| **2** | **Are you a registered user of *Observadores del Mar*?** | |
|  | Yes, I have registered for the training | 47.1 |
|  | Yes. I was already a registered user | 41.2 |
|  | No | 11.8 |
| **3** | **Was the program clearly presented at the beginning of the workshop (objectives, methodology, duration, etc.)?** | |
|  | 7 above 10 | 5.9 |
|  | 9 above 10 | 11.8 |
|  | 10 above 10 | 82.4 |
| 4 | **Has the information on the application of the sampling protocol been provided clearly?** | |
|  | 9 above 10 | 23.5 |
|  | 10 above 10 | 76.5 |
| **5** | **Evaluate the contents and the technical level of the theoretical part** | |
|  | 8 above 10 | 5.9 |
|  | 9 above 10 | 17.6 |
|  | 10 above 10 | 76.5 |
| **6** | **Evaluate the practical part (organization, development of the training, etc.).** | |
|  | 6 above 10 | 5.9 |
|  | 9 above 10 | 23.5 |
|  | 10 above 10 | 70.6 |
| **7** | **The application of the sampling protocol was** | |
|  | Easy | 100 |
|  | Moderate | 0 |
|  | Difficult | 0 |
| **8 (*)** | **The second sampling was** | |
|  | Easy, as the first time | 75 |
|  | Easier than the first time | 25 |
| **9 (*)** | **In the second sampling, I need** | |
|  | Less time for more colonies | 63 |
|  | Same time for more colonies | 13 |
|  | Do not remember | 24 |
| **10** | **Did you find it convenient to dedicate some time after the sampling protocol performance to comment on the results?** | |
|  | 7 above 10 | 5.9 |
|  | 8 above 10 | 11.8 |
|  | 9 above 10 | 41.2 |
|  | 10 above 10 | 41.2 |
| **11** | **Had you previously noticed gorgonian mortality?** | |
|  | Yes, I have seen it before | 52.9 |
|  | No, I have never noticed | 41.2 |
|  | Do not remember | 5.9 |
| **12** | **What do you think warming mortality on gorgonian will be like 10 years from now?** | |
|  | Higher than now | 100 |
|  | Equal than now | 0 |
|  | Lower than now | 0 |
| **13** | **Do you see yourself capable of repeating the sampling protocol on your own in future dives?** | |
|  | Yes | 100 |
|  | No | 0 |
| **14** | **What is your general satisfaction level with the training?** | |
|  | 8 above 10 | 11.8 |
|  | 9 above 10 | 23.5 |
|  | 10 above 10 | 64.7 |
| **15** | **Have you uploaded the data obtained to the *Observadores del Mar* platform?** | |
|  | Yes | 70.6 |
|  | No | 29.4 |
| **16** | **If so (question 15), how do you rate the data uploading process?** | |
|  | 7 above 10 | 8.3 |
|  | 8 above 10 | 33.3 |
|  | 9 above 10 | 8.3 |
|  | 10 above 10 | 50 |
| **17** | **What is your general assessment of the MCS platform *Observadores del Mar*?** | |
|  | 5 above 10 | 5.9 |
|  | 7 above 10 | 11.8 |
|  | 8 above 10 | 11.8 |
|  | 9 above 10 | 17.6 |
|  | 10 above 10 | 52.9 |
| **18** | **Would you be interested in participating in other trainings of *Observadores del Mar*?** | |
|  | Yes | 94.1 |
|  | No | 5.9 |
| **19 (*)** | **After having conducted the training on two occasions, would you be interested in conducting more sampling protocols in the future and being able to monitor these populations?** | |
|  | Yes | 100 |
|  | No | 0 |

**Table S3.** Taxonomic coverage of the Coral Alert project (citizen science platform *Observadores del Mar*; www.observadoresdelmar.es) expert-validated observations from 2003 to 2022. For each species, the total number of validated observations, including the incidental observations and those that include the Mortality Rapid Assessment protocol; the total number of sampling protocol-validated observations, and the total number of sampled colonies in the sampling protocol observations are reported.

| Species observed by citizen scientists and validated by experts | Number of observations | Number of sampling protocol observations | Number of colonies sampled in the sampling protocol |
| --- | --- | --- | --- |
| *Alcyonium acaule* | 5 | 0 | 0 |
| *Alcyonium coralloides* | 2 | 0 | 0 |
| *Astroides calycularis* | 22 | 0 | 0 |
| *Balanophyllia europaea* | 10 | 0 | 0 |
| *Balanophyllia regia* | 2 | 0 | 0 |
| *Cladocora caespitosa* | 185 | 0 | 0 |
| *Corallium rubrum* | 68 | 1 | 16 |
| *Dendrophyllia ramea* | 1 | 0 | 0 |
| *Eunicella cavolini* | 47 | 8 | 628 |
| *Eunicella singularis* | 241 | 42 | 4,058 |
| *Eunicella verrucosa* | 4 | 0 | 0 |
| *Leptogorgia ruberrima* | 1 | 0 | 0 |
| *Leptogorgia sarmentosa* | 12 | 0 | 0 |
| *Leptosammia pruvoti* | 3 | 0 | 0 |
| *Madracis pharensis* | 1 | 0 | 0 |
| *Oculina patagonica* | 97 | 0 | 0 |
| *Oulastrea crispata* | 1 | 0 | 0 |
| *Paramuricea clavata* | 297 | 120 | 11,787 |
| *Parazoanthus axinellae* | 4 | 0 | 0 |
| *Pelagia noctiluca* | 1 | 0 | 0 |
| *Savalia savaglia* | 7 | 0 | 0 |
| Non - identified | 57 | 0 | 0 |
| **TOTAL** | **1,068** | **171** | **16,489** |
